# Why Is Production of Animal Source Foods Important for Economic Development in Africa and Asia?

**DOI:** 10.1093/af/vfaa036

**Published:** 2020-10-30

**Authors:** Isabelle Baltenweck, Dolapo Enahoro, Aymen Frija, Shirley Tarawali

**Affiliations:** 1 ILRI, Policies Institutions and Livelihoods Program, Nairobi, Kenya; 2 ICARDA, Social, Economy & Policy Research Program, Tunis, Tunisia

**Keywords:** animal-source-foods, employment, income, livelihoods, low-and-middle-income-countries

**Implications**

- Production of animal source foods contributes to economic development by generating income and employment for livestock keepers and actors along livestock value chains.
- In Africa and Asia, their production cannot be analyzed without considering the other functions of livestock: livestock are an asset, a store of wealth for resilience and a factor of production in mixed farms.
- Local livestock production increases the availability of animal source foods as a source of proteins and micronutrients, necessary for a healthy population.
- Livestock provide a pathway to women empowerment and gender equality, which are important factors for economic development.

## Introduction

Public debates over the economic, health, social, and environmental merits of livestock are growing increasingly acrimonious. On one hand, livestock is said to contribute to income and wealth generation, increase overall farm productivity, be a source of foreign revenues, promote women’s empowerment, and improve nutrition and health, especially for the most vulnerable. On the other hand, livestock is blamed for environmental degradation and condemned as a potential source of zoonotic disease. For its critics, small-scale livestock farming is economically unproductive, a relic to be set aside as informed consumer demand moves toward a mostly plant-based diet (and even, perhaps, plant-based meat). Overconsumption of animal source foods may also contribute to poor health outcomes (Yang et al., 2016). The current COVID-19 crisis is only amplifying these debates. The economic consequences of COVID-19 are likely to increase hunger and poverty in the developing world, even as the pandemic demonstrates how catastrophically unprepared the world is to handle zoonoses.

**Figure 1.**
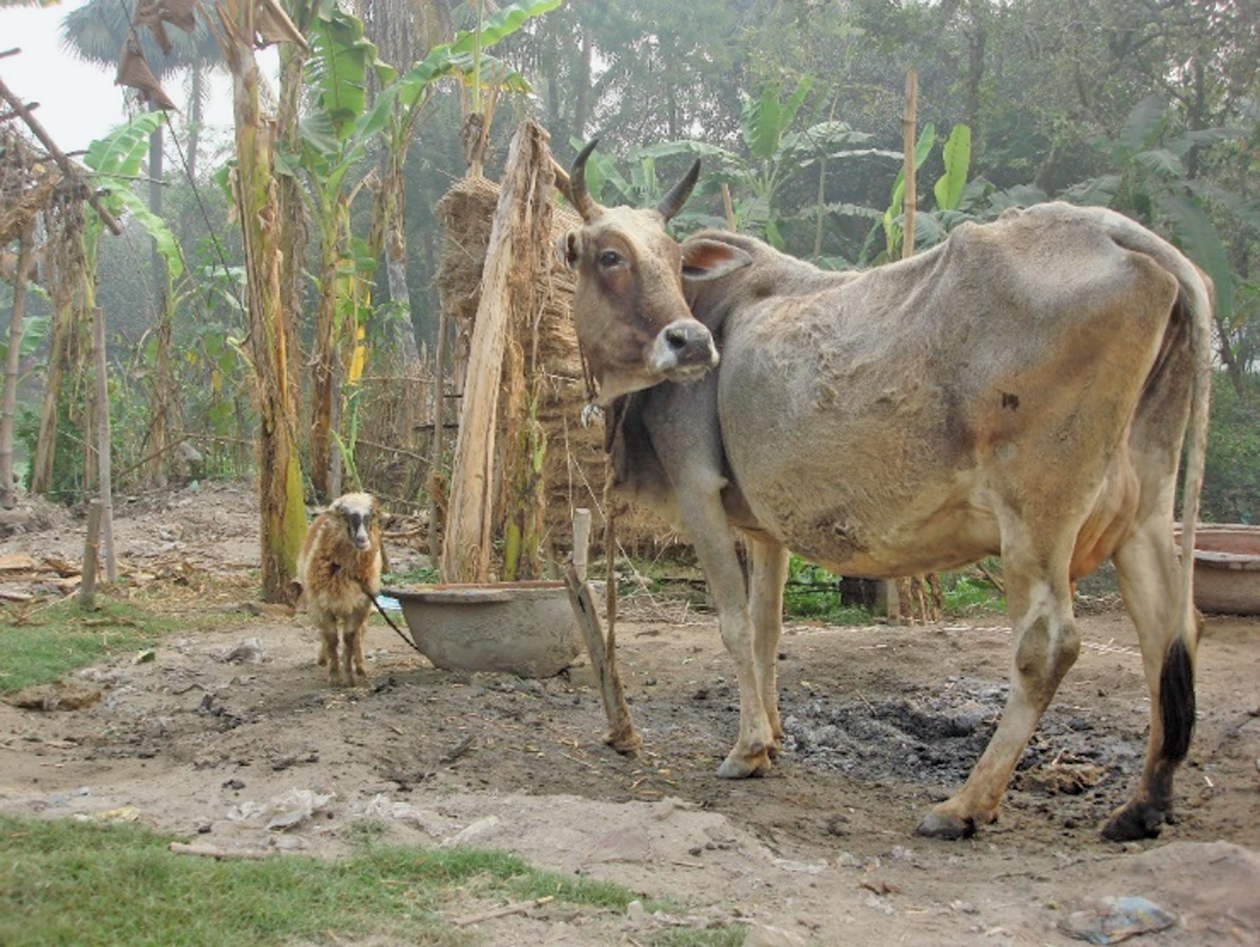
Smallholder livestock household in West Bengal, India (photo credit: ILRI/Susan MacMillan).

To get clarity on this debate, we will look at the relationship between animal source foods production and economic development at the household, community, and national levels. For the purposes of this discussion, animal source foods will exclude fish, which has distinct theoretical and data issues. Our focus is on low- and middle-income countries (see https://data.worldbank.org/income-level/low-and-middle-income for a list) in Africa and Asia where small- and middle-scale farmers produce most of the food (Figure 1). It is important to note that production of animal source foods cannot be done without considering animals being part of a broader system. Livestock functions as an asset and is indicative of household and community prestige.

The paper is organized into four parts: (1) description of the links between livestock production and economic development at the national level, (2) analyzes the current relationship between livestock production and economic development, (3) details the projections for ASF demand in coming decades, and (4) discusses a pathway for meeting demand and what that means for economic development. This paper concludes by determining how the implications of these analysis can impact policy and economic investment.

## Production of Animal Source Foods and Economic Development: What We Know

The simplest, albeit approximate, way of looking at the relationship between livestock production and economic development is through the contribution of livestock to the agricultural gross domestic product (**GDP**). The contribution of livestock to the agricultural GDP varies greatly across countries (LD4D, 2018) averaging ~25% for low- and middle-income countries compared with nearly 50% for high-income countries. This reflects the fact that richer countries have larger and well-coordinated livestock sectors. Limited data are available for agriculture and livestock in low- and middle-income countries and that which is available is often unreliable (Pica-Ciamarra et al., 2015).

**Figure 2.**
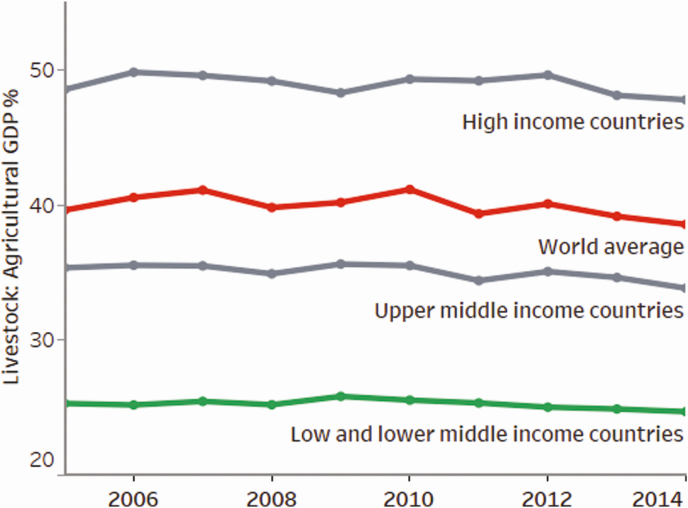
Contribution of the livestock sector to agricultural GDP. *Source*: LD4D (2018).

Based on FAOSTAT data, LD4D (2018) summarized FAOSTAT data revealing that, on average, the richer countries generate a higher share of livestock contribution to the total agricultural GDP (Figure 2). This positive relationship supports the argument that livestock contribute to economic growth. Likewise, it may also be concluded that demand for animal source foods is higher in richer countries, leading to a stronger livestock sector.

**Figure 3.**
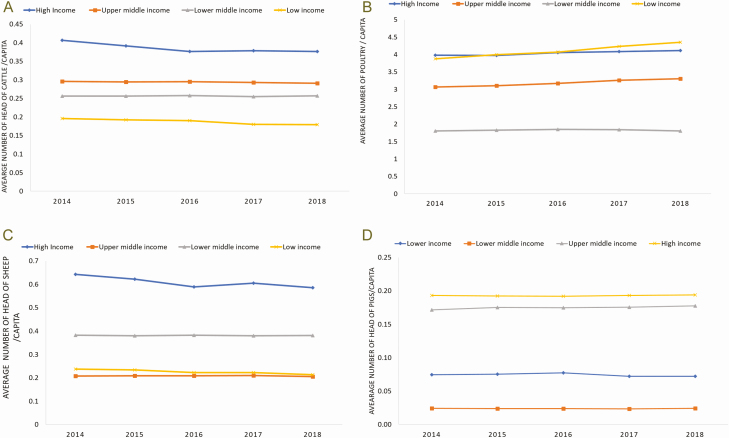
Average number of cattle, poultry, sheep, and pig per capita and income category. *Sources*: authors’ calculations, FAOSTAT (different years). Data on live animals (stocks). http://www.fao.org/faostat/en/#data/QA [assessed June 2020].

That said, there are important differences in terms of species composition as seen in Figure 3. Cattle populations are larger in high-income countries (expressed on a per capita basis) while both high- and low-income countries have large poultry populations. Differences in economic contribution are not only seen across species, but also within species. This explains the poultry figures as local, more resilient poultry genetics are reared with low inputs, but generate low productivity in low-income countries, compared with highly productive animals in the richer countries.

Several authors note that GDP calculations understate the full monetary contribution of livestock to the economies of low- and middle-income countries. For example, Behnke (2010) explained how livestock play a significant role in reducing poverty in rural Sub-Saharan Africa where livestock keepers create high-value products from their animals. At the farm level in Africa and Asia, livestock production generates income through the sale of milk, eggs, and meat, as well as from the sale of products such as wool, hides, and skins. Besides income generation, livestock is used to accumulate and store wealth and in pastoral communities are often the only major asset (Abay and Jensen, 2020). Small and large animals constitute a “‘walking savings account” used to purchase agricultural inputs, as a means to invest in other income-generating activities, or to pay for expenses; planned (education and weddings) and unplanned (medical bills or funeral costs). Livestock production contributes to the economic development at the household and community level through this income and saving function.

Livestock products are consumed on the farms where they are produced or sold. These animal source foods provide proteins and micronutrients to rural or periurban populations where such products are often unavailable and/or expensive. The availability of these animal source foods from on-farm production or purchased from an outside source play a key role in achieving a balanced diet in low- and middle-income countries and are especially important during the first 1,000 d (Iannotti, 2018; GAIN, 2020). By making animal source foods affordable and accessible, local livestock production contributes to a healthy and productive population resulting in almost immediate effects on economic development. The long-term benefits of improved nutrition and well-being of young children include more years of school participation which yields a higher lifetime wage earning, higher adult wages, and increased household assets (Victora et al., 2008). A review of the literature by Halim et al. (2015) suggested that improved maternal and child nutrition interventions (some providing protein rich meals) ultimately resulted in an increase in adult wage income by 46%.

In mixed crop-livestock systems, livestock manure is used to maintain soil fertility and their draft power for ploughing or transport (oxen, buffalos, and horses). In that manner, livestock contribute to whole farm productivity whereby higher crop yields generate more money from a greater quantity of higher quality food. It is evident that animals are key in producing high-quality, high-yielding crops. It is on such mixed farming systems that between 41% and 85% of cereals (maize, rice, sorghum, and millet) are produced in Africa (Thornton and Herrero, 2015).

The economic benefit of the livestock sector reaches beyond the farm by providing employment for individuals associated with the many allied industries throughout the value chain. These allied industries include agricultural inputs and services such as feed, animal health and breeding services, equipment and machinery, and banks and insurance companies that service farmers. In Kenya, the dairy sector employs 3 million people (https://www.kdb.go.ke/) or about 15% of the labor force. These jobs include those in formal settings such as quasi-industrial milk processing plants and abattoirs to jobs purchasing milk from farms and selling it, without processing, to households in small-scale kiosks or on so-called wet, informal markets.

Finally, a point not often recognized is that livestock contribute to economic development through women’s empowerment. Gender equality is widely acknowledged to contribute to economic development. Gender inequality is estimated to be responsible for the loss of 11% of Africa’s total wealth (2014 figure; Wodon and De la Briere, 2018), and livestock play an often unrecognized role in the lives of rural women. Given the gender norms prevailing in many low- and middle-income countries, livestock are one of the few assets that women can own and are a key tool for women’s empowerment. Livestock provide a mechanism to improve women’s income, access information, leverage social networks and provide nutritious food to their families.

At the national level, livestock generate important second-level multiplier effects. Gelan et al. (2012) describe the situation in Ethiopia whereby growth in livestock productivity generates a higher increase in income for all factors of production and in particular from labor compared with other (nonlivestock) commodities, leading to economic growth.

While livestock production contributes to economic development, the effect is also true the other way around. Economic development leads to growth of the livestock sector to meet the increased demand for animal source foods. Although that demand can be met through imports, a growing demand is anticipated to generate at least some increases in national production, including in regions such as sub-Saharan Africa where production system changes are considered to be important adaptation strategies in the face of higher demand for animal source foods and increased climate change (Havlik et al., 2015).

It is also important to acknowledge that livestock production can lead to a reduction in economic growth. Livestock are associated with new and emerging zoonoses, which can cause severe economic harm. In addition, they can cause environmental degradation, and may, if animal source foods are overconsumed, cause health problems. A sustainable livestock transformation is possible and is seen by many as a way to re-ignite economies if the trade-offs of livestock intensification are acknowledged.

## Projected Role of Animal Source Foods for Economic Development

Projections of livestock production vary with economic models, but there is a consensus that demand for animal source foods will increase substantially in Africa and Asia in the coming decades (Valin et al., 2014; Zhou and Staatz, 2016; Desiere et al., 2018). Levels of animal source foods consumption remain relatively low in low- and middle-income countries, and nutritionists agree that increased intake is needed to achieve a balanced diet. In Asia, demand increases will be seen from countries that are yet to reach the peak of what has been termed the livestock revolution; a phenomenon in which demand increases substantially in countries undergoing rapid development (Delgado et al., 2001; ILRI, 2019). By contrast, the predicted growth in demand in Africa will be driven largely by population growth. According to projections from IMPACT, a widely used model of global agricultural and food systems (Robinson et al., 2015), by 2050 the demand for meat (beef, sheep, goat, poultry, and pork combined) could reach around 221 million tonnes (**MTs**) in Asia and 58 million MTs in Africa. Compared to 2010, these projections represent a 77% growth in Asia and a 280% growth in Africa.

**Figure 4.**
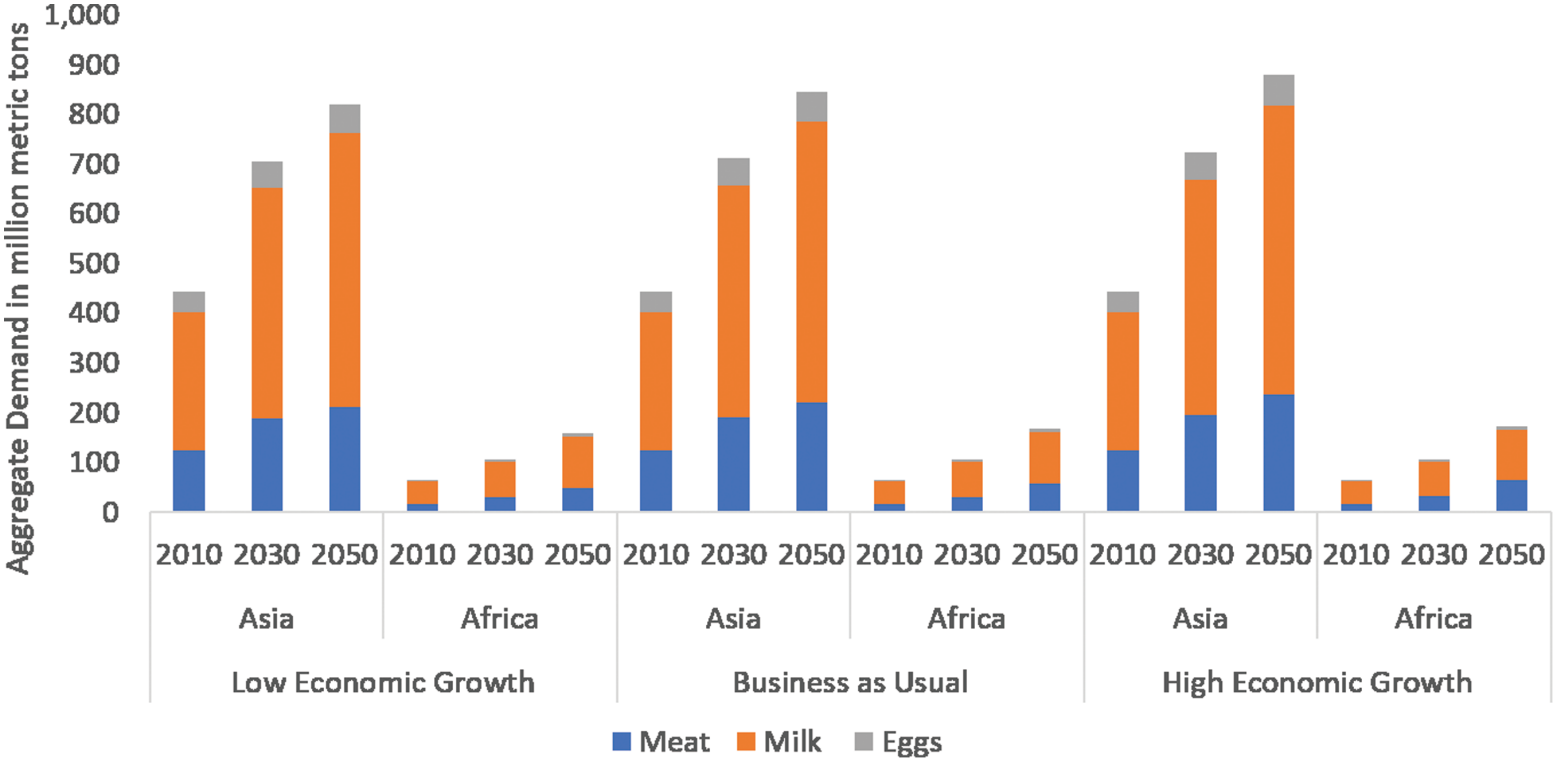
Projections of demand (million metric tons; MT) for animal source foods in Africa and Asia under scenarios of low, medium, and high economic growth from 2010 to 2050. *Source*: authors using IMPACT model version 3.3 (Robinson et al., 2015).

These estimates assume that the key socioeconomic variables driving food demand change will remain constant over the long term. If economic growth exceeds or comes in under expectations (perhaps because of the effects of COVID-19), demand in both regions could rise or falter (Figure 4). (COVID-19 impacts will likely lead to global economic growth that is slower than the recent trends, in which case the “low economic growth” projections become more relevant.) Under these baseline conditions, by 2050, total meat consumption in Asia will account for nearly half (48%) of the global total while; African meat demand will account for 13%. These regions will also see substantial increases in the demand for other animal source foods such as milk and eggs.

**Figure 5.**
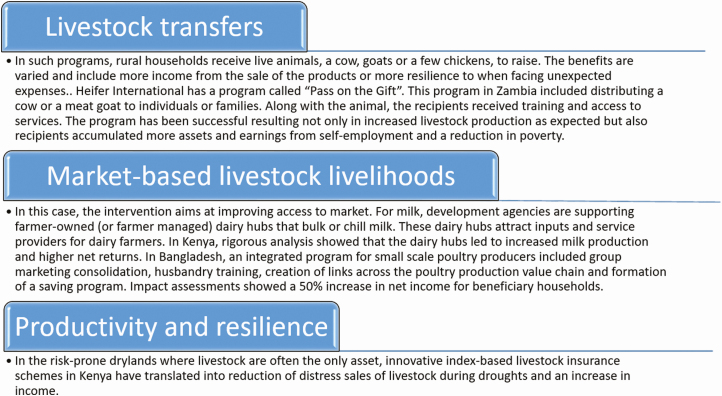
Livestock-related interventions and impact on economic development (based on the study by Staal et al., 2019).

Additionally, livestock-related interventions, activities supported, and financed by development agencies, governments and/or the private sector to improve people’s well-being through livestock, have an impact on economic development. Three types of interventions have been identified by Staal et al. (2019). They are summarized in Figure 5.

The question remains: How can livestock production meet the needs of such an increase in demand and what would be the impact on economic growth? A 2019 paper by ILRI and the World Economic Forum distinguished four possible pathways to meet the growing demand for animal source foods in low- and middle-income countries. These pathways are not mutually exclusive. In likelihood, these pathways (or strategies) will co-exist within a country depending on the product.

**Figure 6.**
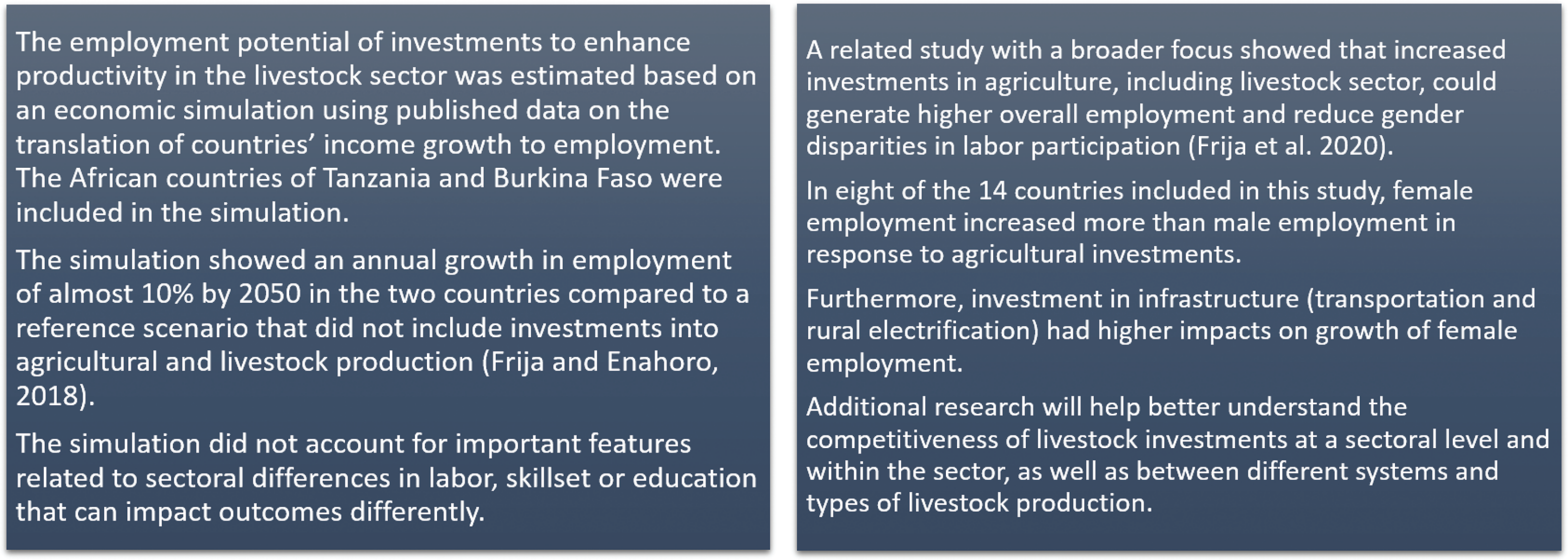
Case study of the employment potential of the livestock sector in Burkina Faso and Tanzania.

The first strategy is to support small- and mid-scale livestock keepers to improve their productivity. Innovations in technologies can help. Better feeding practices, improved genetics, greater access to inputs and services, and better coordination along livestock value chains would translate into greater productivity and higher income. Given the links between livestock production and economic growth, harnessing the growing demand for animal source foods by implementing market-friendly policies, and building better infrastructure could not only improve producer income but also promote economic development at the national level. Changes that enable low-resourced producers and other interested parties to participate in well-functioning livestock value chains could contribute to such economic development being more inclusive (FAO, 2012). A simulation of the employment potential of the livestock sector in two countries in Africa found that well-targeted investments could yield annual growth in employment of 10% a year, with especially positive returns for women (Frija and Enahoro, 2018; Frija et al., 2020) (Figure 6).

The second pathway to meet the increased demand for animal source foods is through the development of industrial-scale livestock production enterprises. Economies of scale can push down production costs and reduce the price of animal source foods. If these large-scale enterprises are integrated with existing livestock enterprises, access to inputs and services may also improve for small- and mid-scale farms. The environmental footprint may be lower as well, due to higher productivity per animal. On the other hand, such production system may displace smaller farms and decrease these farmers’ livelihoods. The net effect on economic development of this second pathway is likely to be positive but not as large as the first pathway. Although a full analysis has not been conducted, the impact of industrialization on family income, employment and women’s empowerment may be nil or negative for some communities.

The third way to increase access to animal source foods is through imports. This pathway can cost jobs on and off livestock farms. Interestingly, an analysis of the dairy sector in West Africa, a region with local production but most of the milk consumed is imported, showed that local milk value chains are growing, generating income and jobs (Corniaux, 2015). Imports are usually more affordable. The cheaper price may result in greater consumption of animal source foods which leads to improved nutrition and ultimately may contribute to higher economic development in the long term.

The last option is to promote alternative proteins. This is a less relevant option in low- and middle-income countries compared with high-income countries, due to availability, costs and cultural preference. In terms of economic growth, these products will generate fewer jobs, compared to those created on farm and along livestock value chains in conventional farming systems.

In summary, the two last pathways provide much lower opportunities for income growth and are unlikely to have significant impacts on economic development.

## Implications for Policies and Investments

The relationship between production of animal source foods and economic development in low- and middle-income countries is complex, works both ways, and operates at different levels. In this paper, we focused on the effects of livestock production on economic development. Richer countries tend to have a larger livestock sector, as indicated by the greater contribution of livestock to their agricultural GDP. At community and household levels in Africa and Asia, livestock contribute to income and employment and are a store of wealth in many settings. Livestock production also impacts economic development through women empowerment and by increasing the availability of nutritious foods. On the other hand, production of animal source foods may also slow down economic development through environmental degradation and the potential for increased human mortality and morbidity due to poor food safety and zoonoses. Livestock makes vital contributions to food and nutrition security and gender equality; these contributions are in addition to the critical direct economic impact provided by livestock and animal source foods.

Policymakers and investors in low- and middle-income countries face complex tradeoffs regarding policy and investments decisions necessary to meet the projected increased demand for animal source foods (Enahoro et al., 2019). Countries with an existing livestock sector should consider supporting small- and medium-scale farmers and value chain contributors to take advantage of the various opportunities offered by livestock and become more profitable and market oriented. More externally orientated pathways to increase supply of animal source foods, such as imports, industrialization, and alternative products, would have a lower impact on economic development and growth in general.

## About the Authors

Isabelle Baltenweck received her MSc and PhD (development economist) from the University of Clermont-Ferrand (France). Working at the International Livestock Research Institute (ILRI) and based in Nairobi, she has 20 yr of postdoctoral experience in agricultural systems in Africa, South and South-East Asia, with a focus on livestock value chains. Over the years, she has increasingly sharpened her skills in gender and social equity research. Working at the interface of research and development, her research has a direct relevance to the activities of development practitioners and the private sector.

Dolapo Enahoro received MS (Applied Economics) and PhD (Regional Science) degrees from Cornell University (USA). She works as a Scientist within the Policies, Institutions and Livelihoods program of the International Livestock Research Institute (ILRI) and is based in Accra, Ghana. She has more than 9 yr of postdoctoral experience in the development and application of analytical frameworks and methodological tools for foresight analysis of livestock systems. In her research, she has applied quantitative foresight approaches and tools in the areas of animal genetics, nutrition and health, markets and livelihoods, the environment, and agricultural and livestock policy in the developing world.

Aymen Frija holds a PhD in agricultural economics from Ghent University (Belgium) and MSc in Agricultural Economics from SupAgro Montpellier (ENSAM) France. He is specialized in economic modelling with focus on natural resources policies and governance. He is currently working at the International Centre for Agricultural Research in the Dry Areas (ICARDA), “Resilient Agricultural Livelihood Systems” program (RALSP). His current research interests include agricultural economic modelling, analysis of farms efficiency and productivity, agricultural water management, institutional performances analysis, and conservation agriculture economics.

Shirley Tarawali is Assistant Director General at the International Livestock Research Institute (ILRI) based in Nairobi, Kenya with responsibilities spanning strategy, communications, and knowledge management as well as institutional management, decision making, and representation. She is also secretary to the ILRI Board of Trustees. Shirley has over 35 yrs’ experience implementing and leading research for development across sub-Saharan Africa and Asia, with a portfolio covering livestock-environment, animal nutrition, and natural resource management. Shirley has authored or co-authored over 50 articles in scientific journal or peer-reviewed publications and contributed in an authorship or editorial capacity to a further 30 plus books and proceedings articles. Shirley holds a PhD in Plant Science from the University of London, UK.
